# Internal incentives for carbon emission reduction in a capital-constrained supply chain: A financial perspective

**DOI:** 10.1371/journal.pone.0287823

**Published:** 2023-07-06

**Authors:** Xiaohui Huang, Juan He, Zhengbo Li

**Affiliations:** 1 School of Transportation and Logistics, Southwest Jiaotong University, Chengdu, Sichuan, PR China; 2 Institute For Supply Chain Finance Studies, National Engineering Laboratory of Application Technology of Integrated Transportation Big Date, Chengdu, Sichuan, PR China; University of Cagliari: Universita degli Studi Di Cagliari, ITALY

## Abstract

Capital constraints hinder enterprises’ carbon reduction efforts and affect the sustainability of the supply chain. To alleviate this limitation, the core enterprise considers offering two financial-based carbon reduction incentive mechanisms: cost-sharing mechanism (CS) and preferential financing mechanism (PF). In a supply chain with the dual sensitivity of market demand to price and carbon reduction, we model each incentive mechanism, discussing their impact, value, and selection strategies. The results show that neither party under CS pursues an excessively high share ratio. Only a below-threshold sharing ratio can promote the supplier’s carbon reduction behavior and improve efficiency for both parties. Conversely, PF has a stable incentive effect on the supplier’s carbon reduction behavior and can effectively increase the retailer’s profits. However, a reasonable carbon reduction standard is needed to attract the supplier. In addition, as market demand becomes more sensitive to carbon reduction, the feasible range of CS narrows and that of PF expands. We compare players’ preferences of PF and CS and find a Pareto region in which all players prefer PF to CS. Finally, we test the robustness of our findings by an extending model. Our study provides guidance for supply chain decisions facing dual pressures of financial constraints and carbon reduction.

## 1. Introduction

As environmental policies and market greening drive, enterprise management and environmental performance become inseparable. The PwC survey found that two-thirds of executives believe that low carbon and environmental protection will play an increasingly important role in global supply chain management. However, in practice, the carbon reduction effect of enterprises is poor, especially for SMEs. In the Sustainability Disclosure Database, SMEs provide only 12 percent of sustainability reports. In China, only 25% of SMEs in the manufacturing sector in Jiangsu province have set up energy management groups to work on low carbon; 176,000 SMEs in Beijing, Tianjin, and Hebei and its surrounding areas were once investigated for pollution. In the era of supply chain competition, this is bound to affect the sustainability of upstream and downstream stakeholders and even the entire supply chain. A clear case exists in the Xiaomi supply chain. As its suppliers were repeatedly disclosed with pollution problems, it nearly caused Xiaomi’s IPO to be blocked. Therefore, in the face of the greening requirements of the supply chain, the carbon emission reduction level of SMEs in the supply chain has gradually become one of the key points of concern for core enterprises.

Some studies have found that financial constraints are one of the major reasons that discourage SMEs from reducing emissions [1]. For example, 40% of Southeast Asian SMEs cited limited financial resources as their greatest barrier to addressing climate change. Therefore, the research questions of our study are: What financial instruments can core enterprises employ to incentivize their capital-constrained partners to increase their investment in carbon reduction? How do different incentive mechanisms operate within low-carbon supply chains? What impact do these mechanisms have on the decisions and benefits of supply chain participants? What conditions must be fulfilled to ensure the feasibility of these incentive mechanisms? When multiple incentives are feasible, how should the supplier and the retailer determine the most suitable one to adopt?

To alleviate the dual pressures of capital constraints and carbon emission reduction in enterprises, large companies in a central position are considering breaking the status quo of small and medium-sized enterprises (SMEs) who are fighting alone in the process of carbon reduction. In recent years, cost-sharing mechanisms (CS) have gained widespread attention from the industry as a funding gap-filling solution to relieve the financial pressure on companies to reduce carbon emissions. Nestle plans to invest 1.2 billion Swiss francs by 2025 to work with suppliers to promote regenerative agriculture. Wal-Mart is also actively involved in green product production and process transformation through CS, including sharing the cost of organic cotton cultivation with suppliers to facilitate the transformation of land from conventional to organic farming [2]. Coca-Cola works with upstream suppliers to innovate emission reduction technologies, use environmentally friendly materials, and significantly reduce carbon emissions through packaging material load reduction.

In fact, another important reason for the onerous financial pressure on SMEs is expensive financing. In contrast, core enterprises usually have better credit standing and can help their smaller partners to obtain lower financing rates by dividing their credit lines. Therefore, in addition to the cost-sharing mechanism, the preferential financing mechanism (PF) with the core enterprises as intermediaries is also a financial innovation method. The basic operation process is as follows: financial institutions, core enterprises, and financing enterprises (SMEs with carbon emission reduction pressure) first sign a tripartite agreement, in which the core enterprises set a carbon emission reduction baseline as the standard for selecting green partners, and small and medium-sized partners commit to paying carbon emission reduction behaviors to meet the standard and apply for preferential interest rate financing from the financial institutions cooperating with the core enterprises. This financing mechanism has been implemented in real-world scenarios due to its ability to provide quick and affordable financing to SMEs. A notable example is the collaborative effort between Walmart and HSBC, which resulted in the establishment of a sustainable supply chain financing program. This program enables suppliers participating in the Gigaton initiative to access preferential financing rates. As part of Walmart’s Project Gigaton, suppliers are required to adopt specific carbon reduction targets. Currently, over 4,500 suppliers have officially enrolled in the project, making it one of the largest private-sector climate action coalitions. The sustainable financing program has proven successful, delivering significant benefits to all involved parties. An additional instance is the partnership between PUMA, a renowned sports brand, and the International Finance Corporation (IFC). IFC offers a more favorable interest rate to PUMA’s suppliers that achieve higher levels of carbon emission reductions. Despite the success of PF in practice, it is still in its infancy in terms of theoretical research, especially from the perspective of supply chain management, which is rarely reported.

In summary, CS and PF as two alternative incentive schemes offered by the retailer, undoubtedly provide two different ways of thinking to promote the development of low-carbon supply chains when financially constrained SMEs have insufficient incentives to reduce carbon emissions. The purpose of this paper is to analyze the mechanism of the CS and PF in a low-carbon supply chain with financial constraints, to describe the interrelationship between the carbon emission reduction incentive effect and economic benefits of supply chain members under different mechanisms, to test their value, and to investigate whether the innovative design of GF is more advantageous in promoting the development of low-carbon supply chains by comparing and analyzing the choice of the two mechanisms. This can not only further enriches the literature on low-carbon supply chains from the financial incentive perspective, but also has positive significance for the promotion and application of green preferential financing incentives in the practice of low-carbon supply chains.

The main work and contributions of this paper can be summarized as follows: (1) Based on the supply chain system where the upstream supplier faces the dual pressure of financial constraints and carbon emission reduction, two carbon emission reduction incentive schemes, CS and PF, are innovatively introduced, and a supply chain decision model with dual sensitivity of price and carbon emission reduction in market demand is constructed respectively. (2) Taking the no carbon emission reduction incentive model as the benchmark, the detailed discussion of functional paths and values of the two incentive mechanisms, and show the impact of demonstrating the feasible regions and related parameters of the two mechanisms. We find that if the sharing ratio is controlled within a certain range, CS can incentivize the supplier to reduce carbon emissions and bring about a small increase in economic benefits for both parties. The incentive effect of PF on the supplier to reduce carbon emissions is constant and can be effective in achieving increased profits for retailers, but reasonable carbon reduction standards need to be set to attract the supplier to join. (3) A comparison of the two incentive mechanisms examines the choice of incentive mechanisms by supply chain players, finds a Pareto region for PF, and verifies the advantages of PF. (4) Establish an extended model with the carbon reduction standard as an endogenous parameter for the retailer, validating the robustness of the findings.

The study is organized as follows. Section 2 reviews the related literature. Section 3 gives the problem description and assumptions. Section 4 builds the benchmark model and two incentive models separately to find the equilibrium solutions in different cases. The action mechanisms of CS and PF are described, and a comparative analysis of CS and PF is performed. Section 5 provides an extended model where the carbon reduction standard is an endogenous parameter for the retailer. Section 6 summarizes the conclusions of this paper and gives suggestions for future research. The detailed proofs of propositions and corollaries in this paper appear in S1 Appendix.

## 2. Literature review

In recent years, many scholars have studied the financing and operation of supply chains with capital constraints from different perspectives, and have achieved rich results. For example, If an upstream supplier faces financial difficulty, he can take advance payment from the downstream retailer [3], or obtain purchase order financing [4, 5] or factoring financing [6] from financial institutions, etc. If a downstream retailer faces financial difficulty, she can apply to the supplier for delayed payment [7–10] or obtain financial support from banks [11, 12]. Some scholars have also studied the issue of bilateral enterprise capital constraints [13] and financing options [14, 15]. A large number of research results have laid a good foundation for effectively solving the capital shortage dilemma of supply chain players, breaking the barrier between finance and the economy. This paper is also based on the background of capital constraint and introduces carbon emission reduction into the supply chain, considering the situation that enterprises face the double pressure of capital constraint and carbon emission reduction requirements.

Some studies have found that technological progress in emission reduction has a positive impact on industrial upgrading, helping to achieve environmental and industrial development [16]. Therefore, how encouraging enterprises to improve their carbon emission reduction level is the focus of our research. At present, a variety of incentives already exist in the real market for enterprises to pay more for carbon reduction behavior. Among them, policy incentives are the most common, such as carbon trading policy [17–23], carbon tax policy [24, 25], carbon standard policy [26, 27], fuel tax policy [28, 29], financial subsidy policy [30–32] etc. These studies systematically described the impact of carbon emission reduction policies, and provide ideas for supply chain players to make a green transformation in different policy contexts. However, in terms of sources, the promulgation of carbon emission reduction policies mainly comes from the government or regulatory bodies, which is a supply chain external incentive. But in the era of supply chain competition, incentives from within the supply chain between upstream and downstream enterprises are equally important.

Some scholars have explored incentives for carbon reduction behavior from the perspective of internal supply chain operations. For example, Rao et al. [33] and Yu et al. [34] suggest that enterprises achieve sustainable development by selecting low-carbon suppliers. The former established a low-carbon supplier selection evaluation index system based on cost, low carbon, quality, and service capability. The latter proposed a green supplier selection model based on carbon footprint to motivate green supply chain participants to make proactive green decisions. Xia et al. [35] investigated the impact of cross-shareholding on operational decisions in low-carbon supply chains with different power structures from a strategic synergy perspective. Yu et al. [36] found that revenue-sharing contracts can achieve low emission levels and low prices through high ratios. Shi et al. [37] pointed out that pure procurement commitment contracts will hinder the carbon abatement performance of the supply chain, but procurement commitment contracts with a carbon abatement target may achieve coordinated improvement of the economic and environmental benefits. Wang et al. [38] investigated the impact of low-carbon advertising on the development of fresh produce supply chains. The above results demonstrate the effectiveness of incentives within the supply chain, but in general, the current research focuses more on the supply chain operations perspective. Unlike their study, although it is also based on internal supply chain incentives, we take into account the financial constraints of the upstream small and medium-sized supplier, and examine how core enterprise can use financial instruments to incentivize the upstream supplier to reduce carbon emissions. We propose two options: a cost-sharing mechanism (CS), and a preferential financing mechanism (PF) that takes into account carbon abatement standards.

At present, CS has received some attention in academia as a means of alleviating pressure on capital costs. Existing studies have addressed various cost-sharing such as advertising inputs [39–42], R&D costs [12, 43], and carbon reduction costs. In particular, in terms of carbon reduction costs, Dai et al. [44] compared two cooperative models, cartel and cost-sharing, in an upstream manufacturer-led green supply chain, and found that the cost-sharing cooperative model could be more profitable for players and lead to peak profits across the supply chain. Yang and Chen [45] validate the role of cost-sharing contracts in the retailer-driven low-carbon supply chain, noting that the cost-sharing contract facilitates system efficiency and leads to the promotion of carbon emission reductions by the manufacturer. Wang et al. [46] developed a single abatement model (one-way cost-sharing contract) and a joint abatement model (two-way cost-sharing contract) respectively, and the results showed that the implementation of both types of contracts can improve carbon emission reduction levels, product volumes, and supply chain profits. Taleizadeh et al. [47] designed different cost-sharing contracts in terms of carbon reduction cost and quality improvement cost, and pointed out the distributor should share the carbon reduction cost rather than the quality improvement cost, as this strategy would not only lead to an improvement in all incentives, but also to price stability. In general, most of the current research on cost-sharing mechanism has been conducted on the basis that supply chain players are well-funded, ignoring the reality that many SMEs are generally under financial pressure in the green transition.

For green financing mechanisms, Huang et al. [48] proposed a green supply chain finance model where interest rates are linked to carbon emission reduction levels when suppliers face financial constraints, emphasizing that the supplier receives decreasing dynamic financing rates as carbon emission reduction levels increase. Qin et al. [49] analyzed the impact of preferential interest rates on enterprises’ carbon emission reduction and found that the interest rate of green finance did not always negatively affect the manufacturer’s carbon emission reduction. Aljazzar et al. [50] discussed the role of deferred payment (trade credit) offered by the supplier to the buyer, and found that deferred payment can improve the environmental and economic performance of supply chains. Zhan et al. [51] considered two ways in which retailers can encourage suppliers’ sustainability practices through advance payments or reverse factoring.

Our research focuses on how core enterprises can use financial instruments to incentivize the carbon reduction behavior of capital-constrained supplier and help them achieve better economic outcomes. We propose two options: (1) a cost-sharing mechanism for the retailer and the supplier to jointly complete carbon reduction investment from the perspective of filling the financing gap; (2) a preferential financing mechanism for the retailer to apply for lower interest rates for the supplier who meet carbon reduction standards from the perspective of reducing financing costs. By constructing a supply chain decision model, the incentive effect, value, and choice of the two mechanisms are explored. The most relevant literature to our study is literature [49], but the current study differs markedly from theirs. First, while literature [49] also considered both CS and GF, they only considered enterprises’ decision power on carbon reduction and did not give supply chain players corresponding pricing power. Intuitively, for different product inputs, enterprises have different pricing schemes or profit levels. Therefore, we return pricing power to the supply chain participants and demonstrate that under a cost-sharing mechanism, when the retailer has pricing power, she transfers some of her costs to the supplier through preferential pricing. Secondly, instead of setting a carbon-reduction standard to select the green supplier, literature [49] has tacitly accepted that preferential rates apply to all suppliers. In contrast, our study explicitly proposes a carbon reduction criterion, which only the supplier who meets this criterion will be eligible for services at a preferential rate. This is designed in the hope that it will directly ensure the incentive effect of carbon reduction. Of course, we also provide insights into how the criteria is set. Finally, literature [49] focus on the phenomenon of underfunding due to the carbon emission reduction investment. In fact, the carbon reduction investment is simply an added financial pressure for most SMEs, who often face financial difficulties at their basic operational stage. In other words, what enterprises lack is not only the cost of carbon abatement investment but possibly also the capital to spend on production. Therefore, we have explicitly captured the two funding gaps, the carbon reduction cost and the production cost, when constructing supply chain decision model to further approximate the actual situation.

## 3. Problem description and assumptions

In a one-to-one supply chain, the supplier is responsible for the production of the product, and wholesales the product to the downstream retailer at price w. The retailer is the core enterprise (supply chain leader) and is responsible for selling the product to the market at the retail price *p*. To focus the analysis on the impact of incentives on enterprise profitability, similar to the literature [47, 52], the retail price is obtained by adding the expected profit to the wholesale price. In addition, carbon emissions occur during the production process. Initial carbon emission per unit product is *e*. As consumers’ awareness of environmental protection increases, consumers pay attention to carbon emission reduction information in addition to the retail price. (Toluna 2019 Sustainability Report shows that 37% of consumers in the survey are willing to pay 5% more for environmentally friendly products.) In the face of the greening trend of the market, the supplier would pay carbon emission reduction level Δ*e* to expand the market with an increment *g*Δ*e*. Therefore, similar to the literature [46, 53], the market demand is *D* = *a* − *bp* + *g*Δ*e*, where *a* is the potential market size, *b* and *g* represents the sensitivity of market demand to the retail price and the carbon emission reduction level respectively. However, carbon emission reduction cost is needed. Without loss of generality, we assume that the corresponding cost is $\frac{1}{2}\eta \Delta e^{2}$, a one-time investment [46, 54]. *η* is the carbon emission reduction cost parameter. *Η* > 0 represents the marginal cost increases with the carbon emission reduction level. In addition to carbon reduction investment costs, the supplier incurs the basic production costs, with the unit production cost denoted by *c*. Considering that low-carbon products can be development-intensive, the impact of carbon reduction behaviors on the unit production cost is very small. For simplicity, drawing on the literature [30, 55, 56], we assume that the unit production cost is unchanged after the implementation of carbon reduction technology. However, the supplier has capital constraints and cannot afford the cost requirements of production and carbon emission reduction. He needs to apply for a loan from the financial institution. Similar to literature [7, 8], we assume that the supplier’s initial fund is zero. The financing amount of the supplier shall be determined according to the carbon emission reduction cost and production cost. However, excessive financing costs can discourage the supplier from investing in carbon emission reduction and affect the sustainability of the entire supply chain. Therefore, the core retailer can incentivize the supplier to reduce carbon emissions through two mechanisms: (a) a cost-sharing mechanism (CS), whereby the retailer shares part of the carbon reduction costs for the supplier to fill part of the funding gap; (b) a preferential financing mechanism (PF), whereby the retailer assist the supplier whose carbon reduction levels meet the standards to apply for more favorable financing rates from the financial institution.

To make it clear, we show the symbols and corresponding notes in Table 1.

**Table 1:** Symbols and corresponding notes.

Symbols	Description
*c*	production cost per unit product
*e*	initial carbon emission per unit product
*a*	initial market size
*b*	sensitivity of market demand to the retail price, *b>0*
*g*	sensitivity of market demand to carbon emission reduction level, *g*>0
*η*	the carbon emission reduction cost parameter, *η*>0
*r*	base financing rate
*s*	the preferential margin of the interest rate
Δ*e*	carbon emission reduction level
*w*	the supplier’s wholesale profit
*m*_*r*_	the retailer’s unit sales profit
*p*	the retailer’s sales profit, *p* = *w* + *m*_*r*_
*π*_*R*_	the retailer’s profit
*π*_*s*_	the supplier’s profit

Also, this article has the following potential assumptions:

Assumption 1: We focus on the dual sensitivity of the demand market to price and carbon reduction levels from the perspective of greening the product market, where market demand can be predicted and regulated based on enterprise pricing and carbon reduction decisions, without considering under- or over-capacity.

Assumption 2: In practice, the supplier’s carbon reduction level can be monitored and verified by an authoritative third-party carbon asset company. Therefore, we tacitly assume that all carbon reduction information committed by enterprises is true.

Assumption 3: All participants in the supply chain have a profit margin and profits must not be negative.

## 4. Model set-up and analysis

### 4.1 Benchmark model

Because SMEs are more likely to face financial difficulties, we assume that the supplier is an SME and obeys the Stackelberg game led by the retailer. At the beginning of the period, the retailer use her leadership advantage to guarantee marginal sales profit *m*_*r*_ first. After catching the retailer’s pricing information, the supplier makes the carbon emission reduction level Δ*e* to expand the market and sells to the retailer at the wholesale price *w* after production is completed. The retailer sells products to consumers at a price *p*, i.e., *p* = *w* + *m*_*r*_.

When the financial institution provides financial support at an exogenous interest rate *r*, the profit functions of the supplier and the retailer can be expressed as follows:

$$\pi_{S}=w(a-bp+g\Delta e)-[c(a-bp+g\Delta e)+\frac{1}{2}\eta \Delta e^{2}](1+r)$$ (1)

$$\pi_{R}=(p-w)(a-bp+g\Delta e)$$ (2)

From Eq (1), the Hessian matrix is $\left[\begin{array}{cc} -2b & g\\ g & -(1+r)\eta \end{array}\right]$, If 2*bη*(1 + *r*) − *g*^2^ > 0, the supplier’s profit function is a concave w.r.t., *w* and Δ*e*. Solving $\frac{\partial \pi_{S}}{\partial \Delta e}=0$ and $\frac{\partial \pi_{S}}{\partial w}=0$, the reflection functions of carbon emission reduction level and wholesale price can be obtained:

$$\Delta e=\frac{g(a-bc-bcr-bm_{r})}{2b\eta (1+r)-g^{2}}$$ (3)

$$w=\frac{\left(1+r)[a\eta +c(b\eta +b\eta r-g^{2})-b\eta m_{r}\right]}{2b\eta (1+r)-g^{2}}$$ (4)

Submitting results to Eq (2), we have $\frac{\partial^{2}\pi_{R}^{}}{\partial m_{r}^{}{}^{2}}<0$, that is, the retailer’s profit is concave in *m*_*r*_. So the optimal marginal sales profit is:

$$m_{r}^{*}=\frac{a-bc(1+r)}{2b}$$ (5)

Submitting results to Eqs (3) and (4), the supplier’s optimal carbon emission reduction level and wholesale price are:

$$\Delta e^{*}=\frac{g[a-bc(1+r)]}{4b\eta (1+r)-2g^{2}}$$ (6)

$$w^{*}=\frac{\eta (1+r)[a-bc(1+r)]}{4b\eta (1+r)-2g^{2}}+c(1+r)$$ (7)

The retail price and market demand are:

$$p^{*}=\frac{a+bc(1+r)}{2b}+\frac{\left(1+r)\eta [a-bc(1+r)\right]}{2[2b\eta (1+r)-g^{2}]}$$ (8)

$$D^{*}=\frac{b\eta (1+r)[a-bc(1+r)]}{4b\eta (1+r)-2g^{2}}$$ (9)

Profits of the supplier and the retailer are:

$$\pi_{S}^{*}=\frac{(1+r)\eta {\left[a-bc(1+r)\right]}^{2}}{8[2b\eta (1+r)-g^{2}]}$$ (10)

$$\pi_{R}^{*}=\frac{(1+r)\eta {\left[a-bc(1+r)\right]}^{2}}{4[2b\eta (1+r)-g^{2}]}$$ (11)

We take this model as a benchmark. It is not difficult to find that the decisions and profits of players are highly related to the interest rate *r*. Financing cost brought by the supplier’s capital constraint reduces the carbon emission reduction level and players’ profits, i.e., $\frac{\partial \Delta e^{*}}{\partial r}<0$, $\frac{\partial \pi_{S}{}^{*}}{\partial r}<0$, $\frac{\partial \pi_{R}{}^{*}}{\partial r}<0$. Therefore, to cater to consumers’ green preferences and increase profits, the retailer can consider providing support to encourage the supplier’s carbon emission reduction behavior. We analyze two carbon emission reduction incentive mechanisms (CS and PF) and explore their value.

### 4.2 Carbon emission reduction incentive model

#### 4.2.1 Model CS

**Fig 1:**
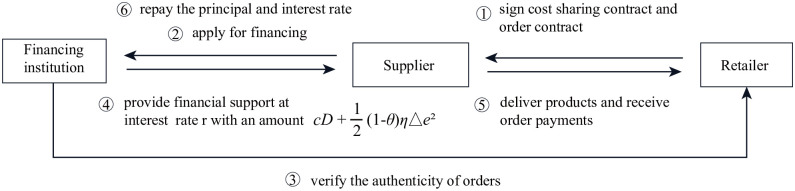
The relationship between participants under CS.

Under CS, the retailer agrees to share *θ* portion of the carbon abatement cost for the supplier, and the supplier only has to bear the remaining (1-*θ*) portion, thus filling the funding gap and alleviating the financial pressure on the supplier. The sequence of events (1) the retailer gives a sharing ratio *θ* to the supplier; (2) the supplier decides to agree or reject the retailer’s cost-sharing; (3) If the supplier accepts, the retailer will sign a cost-sharing contract with him and determine the marginal sales profit $m_{r}^{CS}$; (4) the supplier decides on carbon emission reduction level Δ*e*^*CS*^ and wholesale price *w*^*CS*^ to influence market demand and complete production; (5) when market demand is realized, the retailer purchases on demand and sells to consumers at a retail price *p*^*CS*^, then the supplier repays the principal and interest to the financing institution; (6) If the supplier refuses to accept *θ*, the supplier would still afford all the cost of carbon emission reduction and thus return to the benchmark model. The relationship between participants under CS is shown in Fig 1.

The profit functions of the supplier and the retailer are:

$$\pi_{S}^{CS}=w^{CS}(a-bp^{CS}+g\Delta e^{CS})-[c(a-bp^{CS}+g\Delta e^{CS})+\frac{1}{2}(1-\theta )\eta \Delta e^{CS}{}^{2}](1+r)$$ (12)

$$\pi_{R}^{CS}=(p^{CS}-w^{CS})(a-bp^{CS}+g\Delta e^{CS})-\frac{1}{2}\theta \eta \Delta e^{CS}{}^{2}$$ (13)

With the backward induction approach, we can get the following proposition.

**Proposition 1.** Under CS, if *2bη*(1 + *r*)(1 − *θ)g*^2^ > 0, the supplier’s optimal carbon emission reduction level and wholesale price are:

$$\Delta e^{CS*}=\frac{g(1+r)(1-\theta )[a-bc(1+r)]}{4b\eta {\left(1+r\right)}^{2}{\left(1-\theta \right)}^{2}+2g^{2}[-2+2r(-1+\theta )+3\theta ]}$$ (14)

$$w^{CS*}=\frac{\eta {\left(1+r\right)}^{2}{\left(1-\theta \right)}^{2}[a-bc(1+r)]}{4b\eta {\left(1+r\right)}^{2}{\left(1-\theta \right)}^{2}+g^{2}[-2+2r(-1+\theta )+3\theta ]}+c(1+r)$$ (15)

The retailer’s optimal retail price is:

$$p^{CS*}=\frac{a+bc(1+r)}{2b}+\frac{\left[2b\eta {\left(1+r\right)}^{2}{\left(1-\theta \right)}^{2}+g^{2}\theta ][a-bc(1+r)\right]}{2b\{4b\eta {\left(1+r\right)}^{2}{\left(1-\theta \right)}^{2}+g^{2}[-2+2r(-1+\theta )+3\theta ]\}}$$ (16)

The market demand is:

$$D^{CS*}=\frac{b\eta {\left(1+r\right)}^{2}{\left(1-\theta \right)}^{2}[a-bc(1+r)]}{4b\eta {\left(1+r\right)}^{2}{\left(1-\theta \right)}^{2}+g^{2}[-2+2r(-1+\theta )+3\theta ]}$$ (17)

The profits of the supplier and the retailer are:

$$\pi_{S}^{CS*}=w^{CS*}D^{CS*}-[cD^{CS*}+\frac{1}{2}(1-\theta )\eta \Delta e^{CS*}{}^{2}](1+r)$$ (18)

$$\pi_{R}^{CS*}=\frac{\eta {\left(1+r\right)}^{2}{\left(1-\theta \right)}^{2}{\left[a-bc(1+r)\right]}^{2}}{8b\eta {\left(1+r\right)}^{2}{\left(1-\theta \right)}^{2}+2g^{2}[-2+2r(-1+\theta )+3\theta ]}$$ (19)

Proposition 1 shows that both the retailer and the supplier adjust decisions with sharing ratio *θ* after signing a cost-sharing contract. The change in the decision will further affect the market demand and participants’ profits.

**Corollary 1.** (1) When $\text{0}\leq \theta \leq 1-\frac{g}{2\sqrt{b\eta }(1+r)}$, we have $\frac{\partial \Delta e^{CS*}}{\partial \theta }\geq 0$; otherwise, $\frac{\partial \Delta e^{CS*}}{\partial \theta }<0$. When $\text{0}\leq \theta \leq \frac{1+2r}{3+2r}$, we have $\frac{\partial D^{CS*}}{\partial \theta }\geq 0$; otherwise, $\frac{\partial D^{CS*}}{\partial \theta }<0$.

(2) When $\text{0}\leq \theta \leq 1-\frac{g^{2}}{4b\eta {\left(1+r\right)}^{2}}$, we have $\Delta e^{CS*}\geq \Delta e^{*}$; otherwise, $\Delta e^{CS*}<\Delta e^{*}$. When $\text{0}\leq \theta \leq \frac{\text{1+2}r}{2(1+r)}$, we have $D^{CS*}\geq D^{*}$; otherwise, $D^{CS*}<D^{*}$.

With Corollary 1, we make an interesting discovery: the supplier’s carbon emission reduction level does not continue to increase with sharing ratio. An excessive sharing ratio has a negative impact on carbon emission reduction. Only when the sharing ratio is controlled within a certain region, CS can motivate the supplier to pay a higher carbon emission reduction level. This differs from the results of the study of Qin, Zhao, and Xia (2018). In their study, CS always provides an incentive for enterprises to pay a higher level of carbon emission reduction when the price is exogenous. Instead, our study finds that if the pricing power is given to the supply chain members, the retailer will transfer part of the costs to the supplier by priority pricing of the dominant position. At this point, the supplier’s enthusiasm for carbon emission reduction decreases as sharing ratio increases. At the same time, the market demand, affected by the change of decisions, increases first and then decreases as the sharing ratio increases. Eventually, the advantage accumulated by CS in terms of carbon reduction and market demand disappears when the sharing ratio exceeds a certain threshold.

**Proposition 2.** CS can provide a cooperation region that coordinates both sides.

Proposition 2 shows that the setting of sharing ratio directly affects the feasibility of CS. For both the retailer and the supplier, a high sharing ratio is not the optimal choice. The sharing ratio has a dual impact on enterprise profits. When the sharing ratio is at a low level, CS, because of the improvement of carbon emission reduction level and market demand, can provide a positive profit space to both the supplier and the supplier. With the sharing ratio increase, the retailer increases the retail price to pressure the supplier. This damps the supplier’s enthusiasm for reducing carbon emissions and decreases the market demand although more of the carbon emission reduction cost was transferred. The profits of players will be affected. Therefore, in conjunction with the findings of Corollary 1, business managers should take into account the influence of pricing power allocation on CS practices and exercise caution when determining the sharing ratio. It is crucial to maintain a sharing ratio that falls within an acceptable range, thereby fostering a harmonious relationship between the parties involved. This approach will incentivize the supplier to reduce carbon emissions and meet market demands while ensuring maximum profitability for all participants.

**Fig 2:**
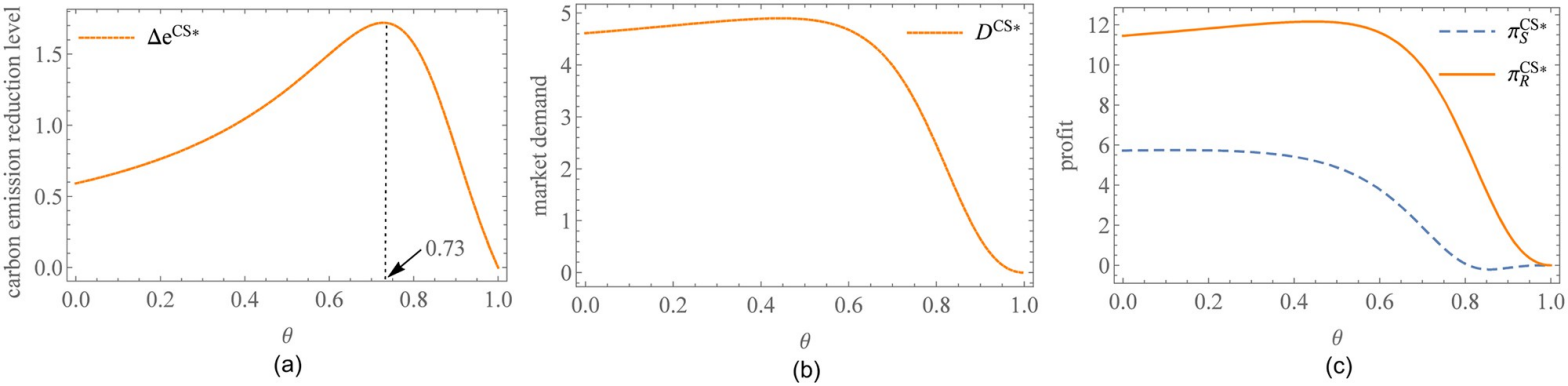
Impact of *θ* on enterprises operation and profits under CS. (a) Impact of *θ* on the carbon emission reduction level. (b) Impact of *θ* on market demand. (c) Impact of *θ* on the profits of the supplier and the retailer.

To visually show the impact of the sharing ratio on carbon emission reduction level, market demand, and enterprise profits, a numerical example is given. Considering practical experience, we determined the parameter values in such a way that the profit and market demand of the participants are not negative. Numerous data sets can meet these criteria, but due to space limitations, we select the following set of data for presentation: *a* = 50, *c* = 9, *η* = 6, *b* = *g* = 3. The detailed information on data description was stated in S1 File. We get some effective information from Fig 2. When *θ* = 0, the supplier will afford all carbon emission reduction costs, which is equivalent to the benchmark model. In Fig 2(a), the carbon emission reduction level of the supplier increases within a certain range and reaches the maximum at *θ* = 0.73. After that, the increase in *θ* damps the supplier’s enthusiasm for reducing carbon emissions until the incentive advantages of CS were consumed, even causing the supplier suspends carbon emission reduction behaviors. Fig 2(b) shows that when *θ* at a low level, market demand has a slowly increasing trend along with *θ* because of the carbon emission reduction level. If *θ* exceeds a threshold, the increase in sharing ratio begins to impact market demand negatively. Fig 2(c) shows the trends of supplier and retailer profits with *θ*. Compared with *θ* = 0, the profits of the supplier and the retailer can increase when *θ* at a low level, but the increment is not huge. This shows that CS does have a cooperative interval that coordinates the two parties. However, the main role of CS is incentives for the supplier to reduce emissions rather than a significant increase in profits. In addition, comparing the feasible regions of the supplier and the retailer, it is not difficult to find that the retailer has a broader space.

**Fig 3:**
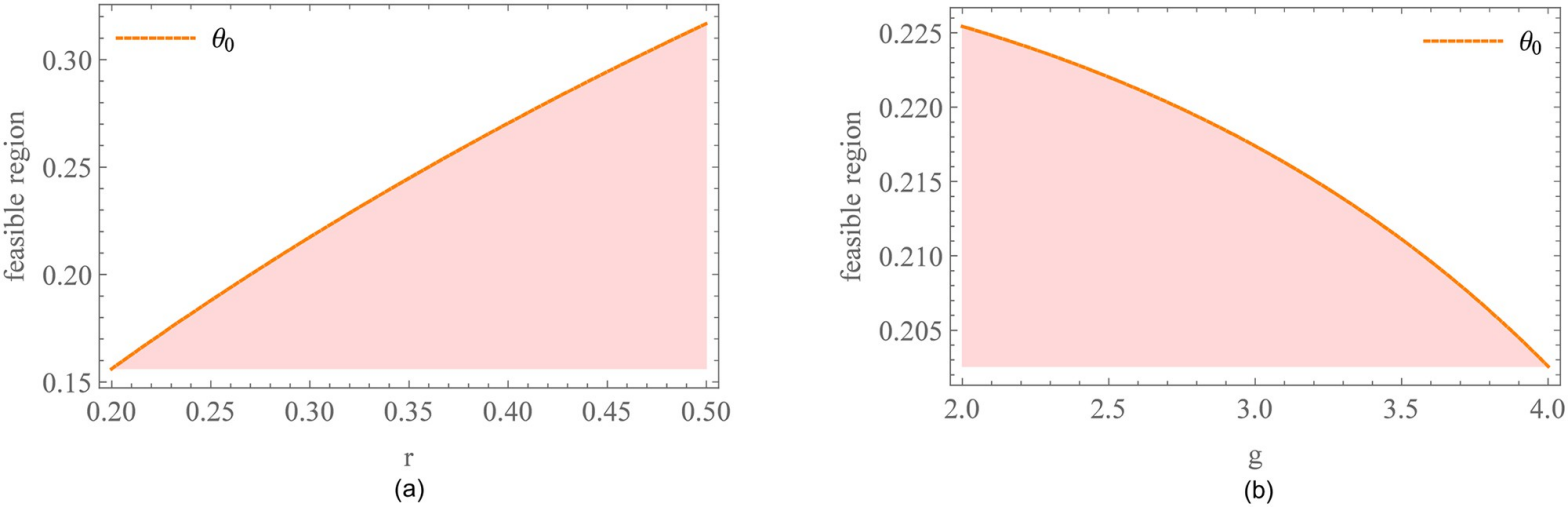
Feasible region of CS. (a) Impact of the interest rate on the feasible region of CS. (b) Impact of the sensitivity of market demand to carbon emission reduction level on the feasible region of CS.

Fig 3 shows the impact of relevant parameters on the feasible region of CS. *θ*_0_ is the feasibility threshold (see Proposition 2). In Fig 3(a), the feasible region expands with the interest rate *r*. This is consistent with the intuition that CS is easier to be accepted in the supply chain with high financing costs. Fig 3(b) presents intriguing insights, indicating that the sensitivity of market demand to carbon emission reduction level has a negative impact on the feasible region of CS. The possible explanation for this observation is that in supply chains with higher carbon sensitivity, the supplier tends to increase his efforts in carbon reduction, resulting in more pronounced fluctuations in market demand. The supplier faces a significant surge in financing costs, prompting him to seek relief. However, the retailer resists bearing excessively high carbon reduction costs and exploits her advantageous pricing power to transfer these costs back to the supplier. This intensifies the conflict in benefit distribution between the two parties, thereby diminishing the consistency of participants’ engagement in CS. Therefore, CS is better suited for supply chains that exhibit lower sensitivity to carbon emission reduction. In such scenarios, the conflict in benefit distribution is mitigated, enhancing the feasibility and effectiveness of CS implementation.

#### 4.2.2 Model PF

**Fig 4:**
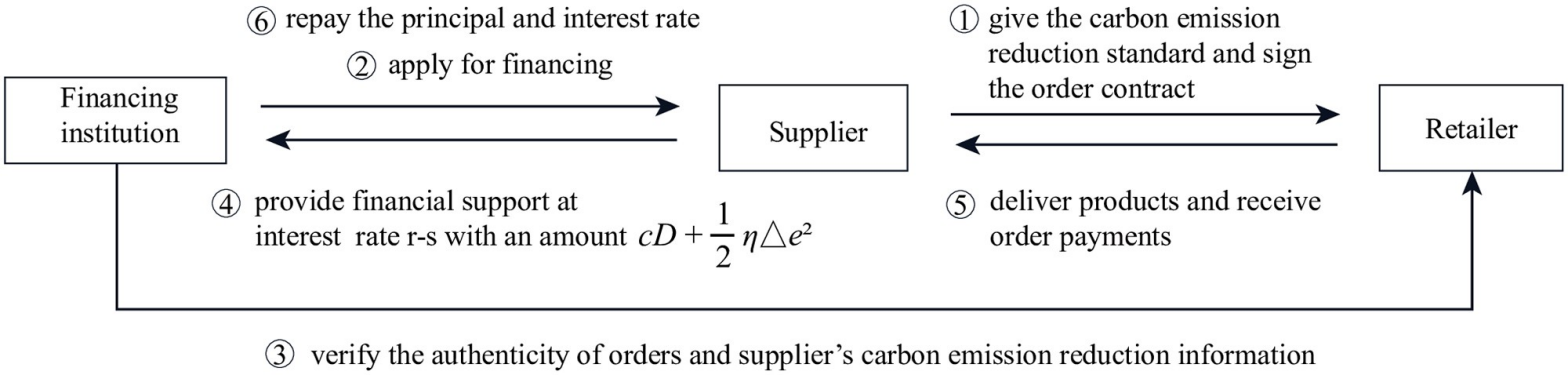
The relationship between participants under PF.

Under PF, the retailer set a carbon reduction standard for the supplier. When a capital-constrained supplier takes out a loan from a financial institution, the retailer can use her credits to assist the supplier who meets the carbon reduction standard to obtain a more favorable financing rate. The preferential margin of the interest rate is *s*. The sequence of events: (1) the retailer gives the carbon emission reduction standard $\underline{e}$; (2) the supplier decides to agree or reject the standard; (3) If the supplier agrees to accept it, the retailer secures a preferential margin of the interest rate *s* (the interest rate becomes *r* − *s* and 0 ≤ *s* ≤ *r*) for him and determine the marginal sales profit $m_{r}^{PF}$; (4) the supplier decides on carbon emission reduction level Δ*e*^*PF*^ and wholesale price *w*^*PF*^ to affect market demand; (5) market demand is realized, the retailer purchases on demand and sells to consumers at a retail price *p*^*PF*^ then the supplier repays the principal and interest to the financing institution; (6) If the supplier refuses to accept $\underline{e}$, it will return to the benchmark model. The relationship between participants under PF is shown in Fig 4.

The profit functions of the supplier and the retailer are:

$$\begin{array}{c} \pi_{S}^{PF}=w^{PF}(a-bp^{PF}+g\Delta e^{PF})-[c(a-bp^{PF}+g\Delta e^{PF})+\frac{1}{2}\eta \Delta e^{PF}{}^{2}](1+r-s)\\ \begin{array}{cc} s.t. & \Delta e^{PF}\geq \underline{e} \end{array} \end{array}$$ (20)

$$\pi_{R}^{PF}=(p^{PF}-w^{PF})(a-bp^{PF}+g\Delta e^{PF})$$ (21)

**Proposition 3.** Under PF, the optimal decisions of players are related to the relationship between preferential margin of interest rate s and carbon emission reduction standard $\underline{e}$, let $s\prime =1+r-\frac{ag+2g^{2}\underline{e}}{bcg+4b\eta \underline{e}}$:

(1) If *s*′ < *s* ≤ *r*, the supplier’s optimal carbon emission reduction level and wholesale prices are:
$$\Delta e^{PF*}=\frac{g[a-bc(1+r-s)]}{4b\eta (1+r-s)-2g^{2}}$$ (22)
$$w^{PF*}=\frac{\eta (1+r-s)[a-bc(1+r-s)]}{4b\eta (1+r-s)-2g^{2}}+c(1+r-s)$$ (23)
  The retailer’s optimal retail price is:
$$p^{PF*}=\frac{a+bc(1+r-s)}{2b}+\frac{\left(1+r-s)\eta [a-bc(1+r-s)\right]}{2[2b\eta (1+r-s)-g^{2}]}$$ (24)
(2) If 0 ≤ *s* ≤ *s′*, the supplier’s optimal carbon emission reduction level and wholesale prices are:
$$\Delta e^{PF*}=\underline{e}$$ (25)
$$w^{PF*}=\frac{g\underline{e}+[a-bc(1+r-s)]}{4b}+c(1+r-s)$$ (26)

The retailer’s optimal retail price is:

$$p^{PF*}=\frac{3a+bc(1+r-s)+3g\underline{e}}{4b}$$ (27)

The market demand and players’ profits are related to the optimal decisions.

If *s′* < *s ≤ r*, the market demand is:

$$D^{PF*}=\frac{b\eta (1+r-s)[a-bc(1+r-s)]}{2{\left[2b\eta (1+r-s)-g^{2}\right]}^{2}}$$ (28)

The profits of the supplier and the retailer are:

$$\pi_{S}^{PF*}=\frac{(1+r-s)\eta {\left[a-bc(1+r-s)\right]}^{2}}{8[2b\eta (1+r-s)-g^{2}]}$$ (29)

$$\pi_{R}^{PF*}=\frac{(1+r-s)\eta {\left[a-bc(1+r-s)\right]}^{2}}{4[2b\eta (1+r-s)-g^{2}]}$$ (30)

If *0 ≤* s *≤* s′, the market demand is:

$$D^{PF*}=\frac{1}{4}[a-bc(1+r-s)\text{+}g\underline{e}]$$ (31)

The profits of the supplier and the retailer are:

$$\pi_{S}^{PF*}=\frac{{\left[a-bc(1+r-s)+g\underline{e}\right]}^{2}-8b\eta {\underline{e}}^{2}(1+r-s)}{16b}$$ (32)

$$\pi_{R}^{PF*}=\frac{{\left[a-bc(1+r-s)+g\underline{e}\right]}^{2}}{8b}$$ (33)

Under PF, the optimal decisions and profits of players are related to the preferential margin of interest rate *s*. For a given carbon emission reduction standard $\underline{e}$, the supplier will be constrained by it when *s* at a low level. He can only increase the carbon emission reduction level to $\underline{e}$ in exchange for a preferential interest rate. If *s* exceeds a threshold, the standard will lose its constraint. The supplier can make his carbon emission reduction decision according to market and operation parameters.

For ease of description, we use *e*′ to denote the carbon emission reduction decision function when the carbon emission reduction standard does not work, i.e., $e\prime =\frac{g[a-bc(1+r-s)]}{4b\eta (1+r-s)-2g^{2}}$. Compared with the benchmark model, the value of PF is shown in Proposition 4.

**Proposition 4.** PF can always encourage the supplier to improve the carbon emission reduction level.

It can be seen from Proposition 4 that the incentive effect of PF on the carbon emission reduction level always exists whatever the carbon emission reduction standard is, even no standard. However, it affects the profits of supply chain members and then affects the feasibility of PF. Therefore, the retailer should set the carbon emission reduction standard reasonably to ensure both sides can reach a consensus on PF.

**Proposition 5.** Let *e*^*PF*^ be the unique value of $\underline{e}$ that satisfies $\pi_{S}^{PF*}-\pi_{S}^{*}=0$. Only if $\text{0}\leq \underline{e}\leq \text{max}\{e\prime ,e^{PF}\}$, both the supplier and the retailer are willing to accept PF.

Proposition 5 illustrates that the setting of the carbon emission reduction standard affects the feasibility of PF. An excessive standard brings heavy pressure on carbon emission reduction to the supplier, leading to a substantial increase in investment capital required for carbon emission reduction. The positive impact generated by the preferential interest rate becomes insufficient to offset the elevated cost increment associated with carbon emission reduction. Consequently, the supplier may decline to accept PF. In this scenario, the retailer must consider the operational capacity and financial condition of the supplier and establish a reasonable carbon reduction standard to enhance the attractiveness of PF for the supplier.

**Fig 5:**
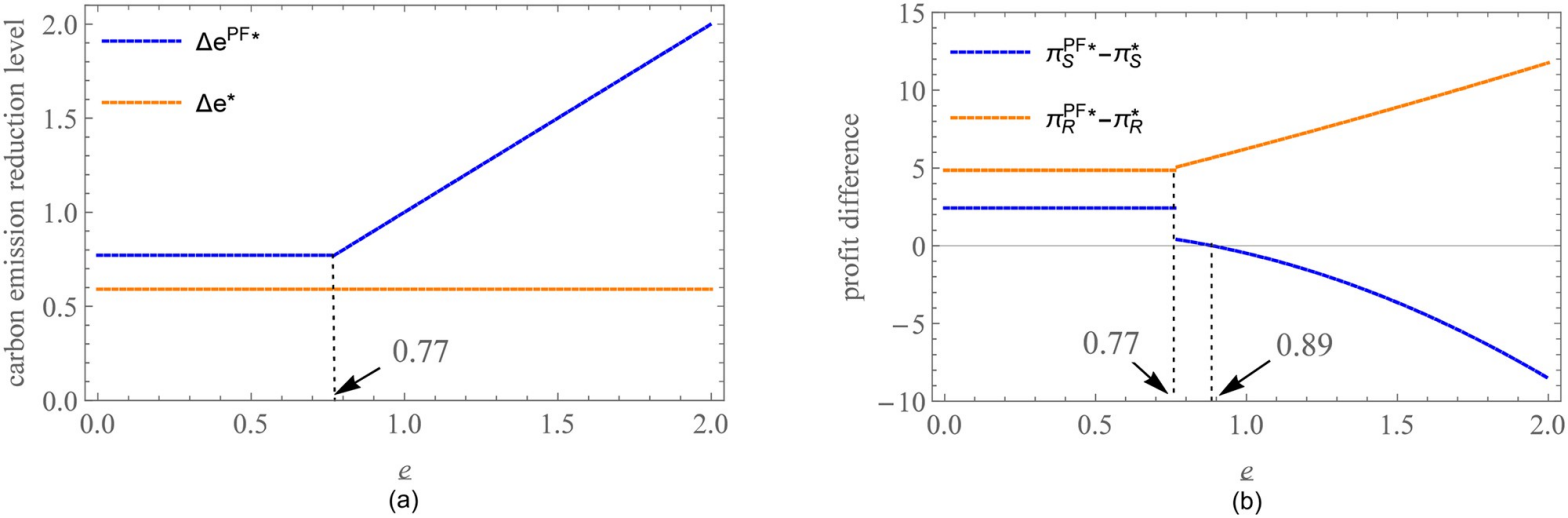
Impact of $\underline{e}$ on enterprises decision and profits under PF. (a)Impact of $\underline{e}$ on the carbon emission reduction level. (b)Impact of $\underline{e}$ on profit difference.

We assume the initial interest rate *r* = 0.3 and the preferential margin of interest rate *s* = 0.1. Fig 5 shows the impact of the carbon emission reduction standard on enterprises decision and profits. Fig 5(a) exhibits that for a given preferential margin of interest rate, the carbon emission reduction standard does not restrict the supplier’s decision if it is at a low level ($\underline{e}<0.77$), which we call the supplier’s active emission reduction space. With the improvement of the carbon emission reduction standard ($\underline{e}\geq 0.77$), the supplier has to increase his carbon emission reduction level to obtain a preferential interest rate. At this time, we call the supplier’s passive emission reduction space. However, no matter how the carbon emission reduction standard changes, the supplier’s carbon emission reduction level is always higher than that of the benchmark model. In other words, the financing cost reduction brought by PF is constant and feasible for enterprises’ carbon emission reduction incentives. Fig 5(b) shows the impact of the carbon emission reduction standard on profit difference. Compared with the benchmark model, both the supplier and the retailer can achieve profit improvement if $\underline{e}<0.77$. Conversely, if $\underline{e}\geq 0.77$, the supplier’s carbon emission reduction decision mode changes, the profit has fallen precipitously and keeps decreasing with $\underline{e}$. Finally, the profit advantage brought by the preferential interest rate to the supplier disappears at $\underline{e}=0.89$, and thus the supplier refuses to accept PF. Conversely, the retailer is willing to set a higher carbon emission reduction standard. The higher the carbon emission reduction standard, the more revenue the retailer can get. Of course, the retailer should also ensure that the supplier can be profitable. So, the retailer must set the carbon emission reduction standard within the feasible range to implement PF smoothly.

**Fig 6:**
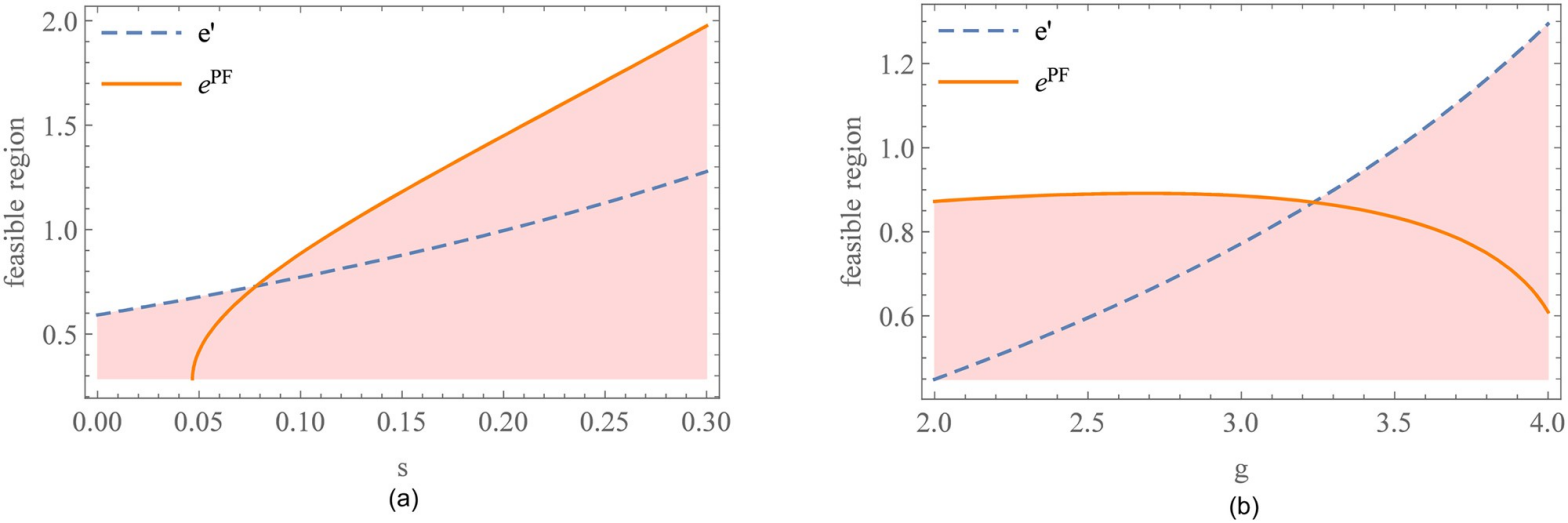
The feasible region of PF. (a) Impact of the preferential margin of interest rate on the feasible region of PF. (b) Impact of the sensitivity of market demand to carbon emission reduction level on the feasible region of PF.

In addition, we further analyze the impact of the preferential margin of interest rate *s* and the sensitivity of market demand to carbon emission reduction level *g* on the feasible region of PF. It can be found from Fig 6(a) that the feasible region expands with *s*. When *s* is at a low level, the carbon emission reduction standard should not exceed the supplier’s active emission reduction space. Otherwise, the constraint of the high emission reduction standard makes the supplier’s profit fall sharply, and thus suffer profit losses. With the increase of *s*, the carbon emission reduction standard has room to play. The larger the *s*, the wider the feasible region of PF. From the perspective of the sensitivity of market demand to carbon emission reduction level in Fig 6(b), when consumers pay more attention to carbon emission reduction information, the supplier will actively increase carbon emission reduction level according to market tendency. The retailer does not have to set a high carbon emission reduction standard to restrict the supplier’s decisions. The larger the *g*, the higher the supplier’s carbon emission reduction level, the faster the feasible region expands. Overall, the feasible range of PF widens with an increase in the preferential margin of interest rate or greater carbon sensitivity in the consumer market. Managers can enhance the implementation of PF by increasing the preferential interest rate margin and promoting consumer awareness of carbon reduction through educational and promotional efforts.

### 4.3. Incentive mechanism comparison and selection

When both incentive mechanisms are feasible, the retailer and the supplier need to choose between them. Firstly, we compare the carbon emission reduction level of the supplier under CS and PF to analyze their performance in environmental protection incentives. Then, we compare the profits of supply chain members under two incentive mechanisms and check the condition under which PF is better than CS. Finally, to ensure the synchronization of the chosen preferences of all players, we try to find a Pareto region where all players have a higher profit.

Comparing the carbon emission reduction level under the two incentive mechanisms, we can get the following conclusion.

**Proposition 6.** When *s* ≥ *s*_*e*_, we have $\Delta e^{PF*}\geq \Delta e^{CS*}$, where

$$s_{e}=\frac{[a-bc(1+r)][4b\eta {\left(1+r\right)}^{2}(1-\theta )-g^{2}]\theta }{4ab(1+r)\eta (1-\theta )-4b^{2}c{\left(1+r\right)}^{2}\eta (1-\theta )\theta -bcg^{2}[2+2r(1-\theta )-3\theta ]}.$$

It can be seen from Proposition 6 that when the preferential margin of interest rate is large, PF encourages the supplier in improving the carbon emission reduction level and thus plays a greater role than CS. In particular, when *θ* = 0, we have *s*_*e*_ = 0. In other words, when the supplier pays all the cost of carbon emission reduction, PF can always provide a higher carbon emission reduction level. This verifies the conclusion of Proposition 4 again.

In addition, we try to analyze the Pareto region of PF greater than CS by comparing the profits of players. There is a certain constraint between the carbon emission reduction standard and the preferential margin of interest rate in PF. For a given $\underline{e}$, a low *s* makes the supplier subject to the carbon emission reduction standard, which will disappear as *s* increases to a high level. Similarly, we discuss whether PF is better than CS by analyzing the relationship between $\underline{e}$ and *s*.

**Proposition 7.** When *s* ∈ [max{*s*_*S*1_, *s*_*R*1_, *s*’}, *r*] ∪ [max{*s*_*S*2_, *s*_*R*2_}, *s*’], PF is better for both the retailer and the supplier than CS.

**Fig 7:**
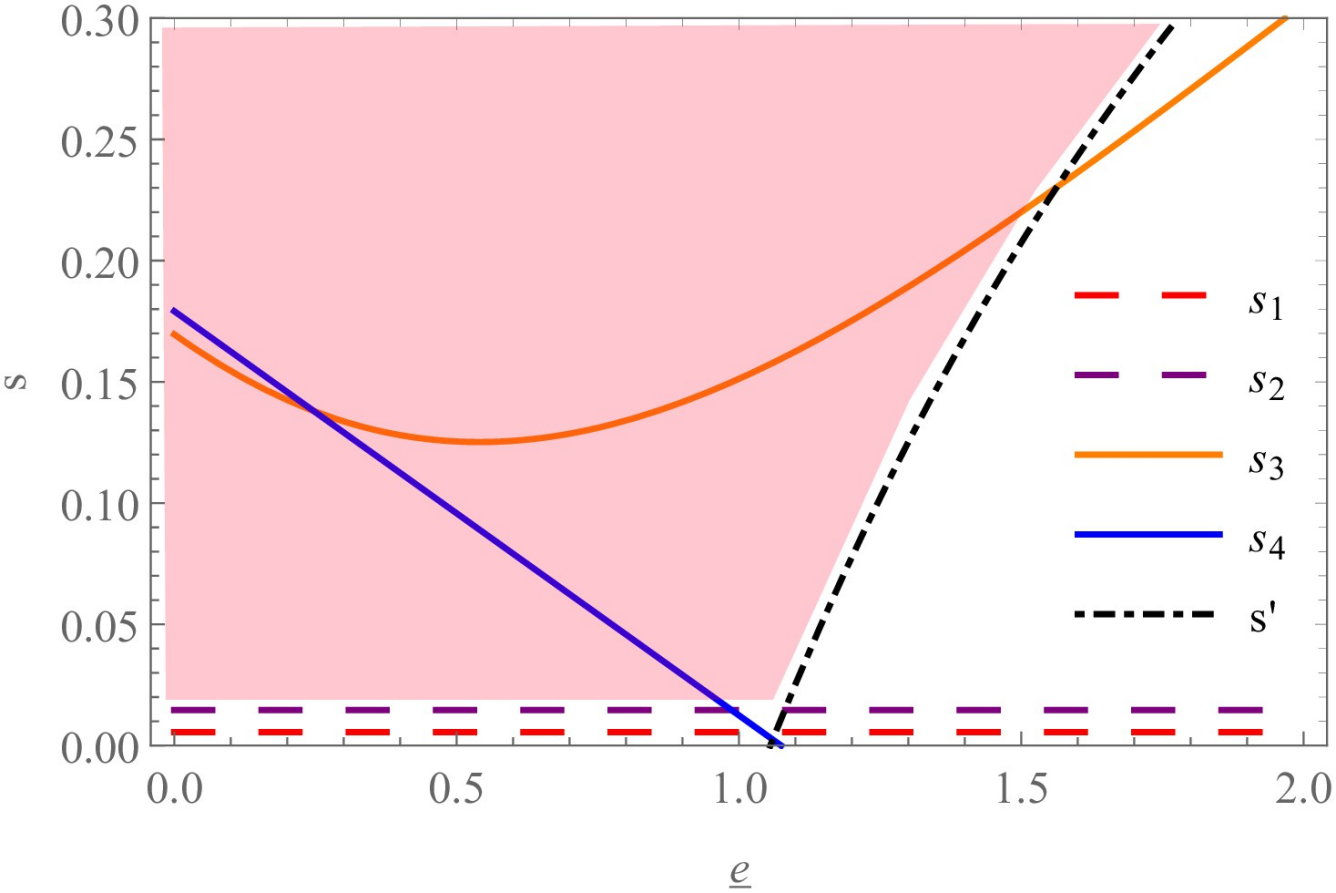
The Pareto region that all players prefer PF over CS.

Proposition 7 shows that there is a Pareto region where players can obtain relatively higher profits under PF in the preferential margin of interest rate *s*. It is not difficult to find that the requirement of *s* in the Pareto region cannot be too low. For visual display, Fig 7 shows the changes in the Pareto region with the carbon emission reduction standard. The dashed line represents the Pareto region boundary when the carbon emission reduction standard has no constraint, the straight line represents the Pareto region boundary when the constraint exists, and the black dot-dashed line represents the boundary dividing whether the emission reduction standard has a constraint. For a low level $\underline{e}$, both parties can reach a consensus on PF when *s* higher than the threshold. With the increase of $\underline{e}$, the Pareto region of PF continues to shrink until it disappears.

## 5. Model expansion

Regarding PF with exogenous carbon reduction standards, we have demonstrated its value in terms of carbon reduction incentives and profit improvement, as well as its advantages compared to CS. We have provided insights into the trade-off between the preferential margin of the interest rate and carbon reduction standards. However, the retailer may also set specific carbon reduction targets based on the operational situation of the supplier, rather than directly adopting existing green enterprise selection criteria. Therefore, in this section, we discuss carbon reduction standards as a decision variable for the retailer to validate the robustness of the model.

**Proposition 8.** When $s\in [0,\hat{s}]\cup [š,r]$, the retailer’s setting of the carbon reduction standard only needs to satisfy $0\leq \underline{e}*<e\prime$. When $s\in (\hat{s},\bar{s})$, $\underline{e}*=e^{PF}$. $\hat{s}$ satisfies $\pi_{S}^{PF*}-\pi_{S}^{*}{|}_{\underline{e}=e\prime }=0$ and $\bar{s}$ satisfies $s\prime -r{|}_{\underline{e}=e^{PF}}=0$.

Proposition 8 indicates that the retailer’s determination of the optimal carbon reduction standards is contingent upon the preferential margin of interest rate offered by financial institutions. When the preferential margin of interest rate is either low or high (i.e., $0\leq s\leq \hat{s}$ or $\bar{s}\leq s\leq r$), the optimal carbon reduction standard is not unique. The retailer simply needs to ensure that the standard falls within the active carbon reduction space of the supplier. However, when the preferential margin of interest rate is at an intermediate level (i.e., $\hat{s}<s<\bar{s}$), the retailer will establish a stable carbon reduction standard to maximize her profit while ensuring that the supplier does not reject PF.

**Fig 8:**
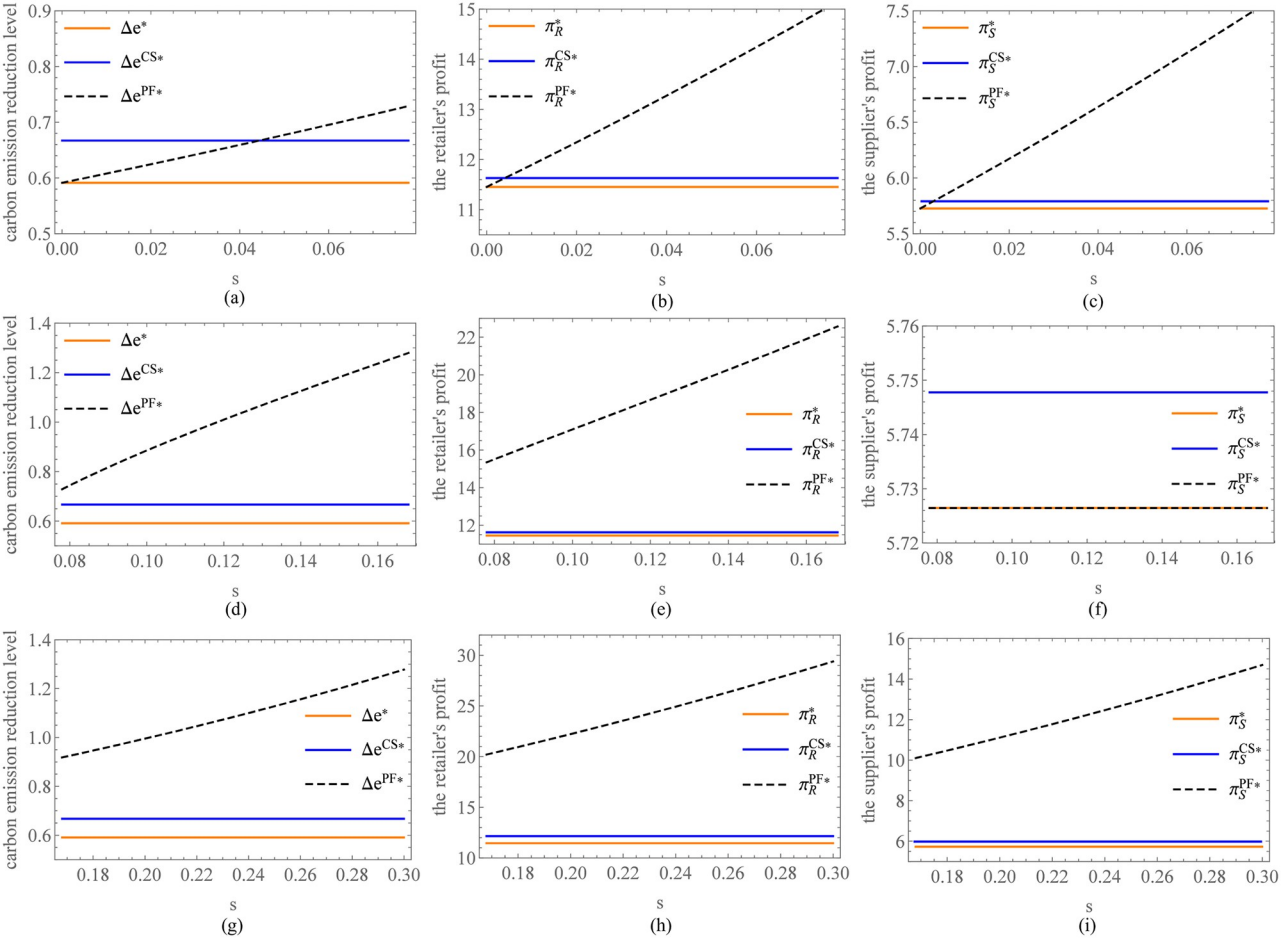
Comparison of the results under different models with different preferential margin of interest rate. (a) Comparison of the carbon emission reduction level. (b) Comparison of the retailer’s profit. (c) Comparison of the supplier’s profit.

In order to more intuitively demonstrate the feasibility and value of PF when the retailer has the authority to set carbon emission reduction standards, we continue with the aforementioned parameter assignment and provide numerical analysis. Through calculations, we can easily obtain $\hat{s}=0.078$ and $\bar{s}=0.168$. According to Fig 3, the CS is feasible as long as the sharing ratio satisfies 0 ≤ *θ* ≤ *θ*_0_. Let’s assume *θ* = 0.1 for the sake of discussion. Fig 8 shows the comparison of the three models (benchmark model, CS, and PF) in terms of carbon emission reduction levels and profit when the preferential margin of interest rate is at different levels. It is evident that compared to the benchmark model, the retailer can effectively incentivize the supplier’s carbon reduction behavior by reasonably setting the optimal carbon reduction standard, thereby improving profits for both parties. Furthermore, compared to CS, it is observed that PF also has a Pareto region when the preferential margin of interest rate falls within a certain range. These findings remain consistent with the results obtained when the carbon emission reduction standard is exogenous, thus validating the robustness of the PF model.

## 6. Conclusion

In this paper, we take an internal supply chain perspective and use financial instruments as an entry point to cost-sharing mechanism (CS) and preferential financing mechanism (PF) into a capital-constrained supply chain to stimulate enterprises’ carbon emission reduction investment. Taking no carbon emission reduction incentive as a benchmark model, we investigate the incentive effect on carbon emission reduction level and economic value of CS and PF, showing when and what incentive schemes could be adopted. To our knowledge, this is the first analysis of PF with a carbon emission reduction standard from a theoretical perspective, and a discussion of the setting of the carbon emission reduction standard line and compare PF with CS. Our analysis yields several findings that may help participants understand the role of different carbon emission reduction incentive mechanisms in low-carbon transformation, and also provides theoretical support for the application of PF in low-carbon supply chain practices.

We mainly get the following insights: (1) CS has a certain incentive effect on carbon reduction, but the supplier’s carbon emission reduction level does not always increase with the sharing ratio. Also from an economic perspective, both parties will only reach a consensus based on CS if the sharing ratio is controlled within a specific range. This is because if the cost-sharing ratio is too high, the retailer as the core enterprise will transfer the cost to the supplier again through the pricing first-mover advantage, which again inhibits the supplier’s willingness to reduce carbon emissions and negatively affects the profits of both parties, and the CS fails. In addition, examining the applicability of CS finds that CS is more likely to be economically valuable in supply chains with high financing costs or in consumer markets that are relatively insensitive to carbon reduction information. (2) Compared to CS, PF has a more stable carbon reduction incentive effect. It can always increase the reduction level of the supplier. From the economic perspective, the feasibility of PF depends on the trade-off between the carbon emission reduction standard and the preferential margin of interest rate. For a given preferential margin of interest rate, the retailer setting a high carbon reduction standard can indeed increase her profits and force the supplier to improve the carbon emission reduction level. However, the supplier only accepts the carbon emission reduction standard below the threshold. The retailer needs to rationalize a carbon reduction standard to attract the supplier. In addition, the higher preferential margin of interest rate or carbon sensitivity in the consumer market can relieve the constraints of carbon reduction standard and expand the feasible region of PF. (3) Comparing the equilibrium decisions and profits of participants of CS and PF, it is found that PF can effectively alleviate the profit conflict between the retailer and the supplier if a reasonable relationship between the preferential margin of interest rate and the carbon emission reduction standard is given. There is a Pareto region where both the retailer and the supplier prefer PF over CS. (4) The extended model of PF, incorporating carbon reduction standards as endogenous parameters for the retailer, is introduced to assess the robustness of the results and find that the main results still hold in the extension.

The research conducted in this paper sheds light on the influence of financial support from core enterprises on decision-making and economic benefits in low-carbon supply chains, offering valuable insights for enterprise management. To implement CS effectively, both parties need to reasonably control the cost-sharing ratio to prevent potential backlash of excessive cost-sharing through the pricing channel. Furthermore, the retailer can utilize PF to incentivize the supplier’s carbon reduction efforts and enhance her own profits, but it is crucial to set reasonable carbon emission reduction standards to attract supplier participation in PF. Additionally, enterprises are better positioned to employ CS in supply chains with higher financing costs or lower carbon sensitivity. The feasibility scope of PF can be expanded by increasing the preferential margin of interest rate or by raising consumer awareness of carbon emission reduction. Finally, enterprises can carefully manage the relationship between the preferential margin of interest rate and carbon reduction standards to maintain consistency in mechanism choice between the two parties.

Although our analysis has achieved some results and management enlightenment, the premise of relevant conclusions for practice is that the retailer has a comprehensive grasp of the upstream supplier’s carbon emission reduction efficiency, production cost, and market information, as well as the sharing of market information such as initial market size, market sensitivity to prices and emission reductions. However, many enterprises in the real world are unwilling to share their information because of the consideration of private information disclosure. The following research can be extended to the study of carbon emission reduction incentives with information asymmetry.

## Supporting information

S1 Appendix (DOCX)

S1 File Data description of numerical examples. (DOCX)
