# Supplementary material for: Internal incentives for carbon emission reduction in a capital-constrained supply chain: A financial perspective
Source: PLoS One. 2023 Jul 6;18(7):e0287823. doi: 10.1371/journal.pone.0287823 (PMC10325093; doi:10.1371/journal.pone.0287823)
Supplement: S1 Appendix — (DOCX) [file pone.0287823.s001.docx]

**Appendix**

**Proof of Proposition 1.**

According to Equation (12) and the first derivative $\frac{\partial\pi_{S}^{CS}}{\partial\Delta e^{CS}}=\frac{\partial\pi_{S}^{CS}}{\partial w^{CS}}=0$, we have $w^{CS}=\frac{cg^{2}(1+r)+\eta(-1+\theta)(1+r)[a+bc(1+r)-bm_{r}]}{2b\eta(1+r)(-1+\theta)+g^{2}}$and$\Delta e^{CS}=\frac{g[-a+bc(1+r)+bm_{r}^{cs}]}{2b\eta(1+r)(-1+\theta)+g^{2}}$. Submitting results to Equation (13), we have , that is, $\pi_{R}^{CS}$ is concave in $m_{r}^{CS}$. When $\frac{\partial\pi_{R}^{CS}}{\partial m_{r}^{CS}}=0$, the optimal marginal sales profit can be obtained: $m_{r}^{CS*}=\frac{[a-bc(1+r)][2b\eta(1+r)^{2}(-1+\theta)^{2}+g^{2}[-1+r(-1+\theta)+2\theta]]}{b[4b\eta(1+r)^{2}(-1+\theta)^{2}+g^{2}[-2+2r(-1+\theta)+3\theta]]}$.

Submitting results to correlation functions to get Proposition 1. □

**Proof of Corollary 1.**

According to Equation (14), we can get that

$\frac{\partial\Delta e^{CS*}}{\partial\theta}=\frac{g(1+r)[a-bc(1+r)][4b\eta(1+r)^{2}(-1+\theta)^{2}-g^{2}]}{[4b\eta(1+r)^{2}(-1+\theta)^{2}+g^{2}[-2+2r(-1+\theta)+3\theta]]^{2}}$.

Thus, when $0\leq\theta\leq1-\frac{g}{2\sqrt{b\eta}(1+r)}$, we have $\frac{\partial\Delta e^{CS*}}{\partial\theta}\geq0$; otherwise, $\frac{\partial\Delta e^{CS*}}{\partial\theta}<0$. That is, the supplier's carbon emission reduction increases first and then decreases in sharing ratio $\theta$. In addition, compared with the benchmark model that no emission reduction incentives, it is found that when $\theta=0$, $\Delta e^{CS*}=\Delta e^{*}$; when $\theta=1$, $\Delta e^{CS*}-\Delta e^{*}=\frac{-g[a-bc(1+r)]}{4b\eta(1+r)-2g^{2}}<0$. There must be a solution satisfied $\Delta e^{CS*}-\Delta e^{*}=0$ within $\theta\in(0,1]$. Let $\Delta e^{CS*}-\Delta e^{*}=0$, we have $\theta=1-\frac{g^{2}}{4b\eta(1+r)^{2}}$. When $0\leq\theta\leq1-\frac{g^{2}}{4b\eta(1+r)^{2}}$, $\Delta e^{CS*}\geq\Delta e^{*}$; otherwise, $\Delta e^{CS*}<\Delta e^{*}$. The proof is similar in the relationship between $D^{CS*}$ and $\theta$. We do not report details of the derivations for conciseness. □

**Proof of Proposition 2**.

We use $\Delta\pi_{S}^{CS}=\pi_{S}^{CS*}-\pi_{S}^{*}$ to indicate the profit changes of the supplier before and after signing a cost-sharing contract. Examining the relationship between $\Delta\pi_{S}^{CS}$ and $\theta$ according to Equations (10) and (18), we find that

$\frac{\partial\Delta\pi_{S}^{CS}}{\partial\theta}=\frac{g^{2}(1+r)^{3}[a-bc(1+r)]^{2}\eta(-1+\theta)^{2}\{4b\eta(1+r)(-1+\theta)[r(-1+\theta)+2\theta]+g^{2}[2r(-1+\theta)+3\theta]\}}{2\{4b\eta(1+r)^{2}(-1+\theta)^{2}+g^{2}[-2+2r(-1+\theta)+3\theta]\}^{3}}$. Assume that $f_{S}(\theta)=4b\eta(1+r)(-1+\theta)[r(-1+\theta)+2\theta]+g^{2}[2r(-1+\theta)+3\theta]$, $\frac{\partial\Delta\pi_{S}^{CS}}{\partial\theta}$ is the same as the positive or negative of $f_{S}(\theta)$. $f_{s}(\theta)$ is convex in $\theta$ because $\frac{\partial^{2}f_{S}(\theta)}{\partial\theta^{2}}=8b\eta(1+r)(2+r)>0$. Let $\frac{\partial f_{S}(\theta)}{\partial\theta}=0$, we can get to the extreme point $\theta=\frac{8b\eta(1+r)^{2}-g^{2}(3+2r)}{8b\eta(1+r)(r+2)}\in[0,1]$, and $f_{S}(\theta)=-\frac{[8b\eta(1+r)-g^{2}(3+2r)]^{2}}{16b\eta(1+r)(2+r)}-\frac{g^{2}r}{\eta(2+r)}<0$ at this point. Besides, there are $f_{S}(\theta=0)>0$ and $f_{S}(\theta=1)>0$ at the boundary point. Therefore, there must be two points satisfied $f_{S}(\theta)=0$ within $\theta\in[0,1]$. When $\theta=0$, $\Delta\pi_{S}^{CS}=0$. When $\theta=1$, $\Delta\pi_{S}^{CS}=-\frac{(1+r)[a-bc(1+r)]^{2}\eta}{8[2b\eta(1+r)-g^{2}]}<0$. Thus, there must be a unique point, $\theta_{0}$, satisfy $\Delta\pi_{S}^{CS}=0$. If $\theta\in[0,\theta_{o}]$, $\Delta\pi_{S}^{CS}\geq0$; if$\theta\in(\theta_{o},1]$, $\Delta\pi_{S}^{CS}<0$. Similarly, we use $\Delta\pi_{R}^{CS}=\pi_{R}^{CS*}-\pi_{R}^{*}$ to indicate the profit changes of the retailer before and after signing a cost-sharing contract. Examining the relationship between $\Delta\pi_{R}^{CS}$ and $\theta$ according to Equations (11) and (19), we find that

$\frac{\partial\Delta\pi_{R}^{CS}}{\partial\theta}=\frac{g^{2}(1+r)^{2}[a-bc(1+r)]^{2}\eta(1-\theta)[1-2r(-1+\theta)-3\theta)]}{2\{4b\eta(1+r)^{2}(-1+\theta)^{2}+g^{2}[-2+2r(-1+\theta)+3\theta]\}^{2}}$.

Assume that $f_{R}(\theta)=1-2r(-1+\theta)-3\theta$, $\frac{\partial\Delta\pi_{R}^{CS}}{\partial\theta}$ is the same as the positive or negative of $f_{R}(\theta)$. we get that $\theta=\frac{1+2r}{3+2r}$ when $f_{R}(\theta)=0$. If $\theta\in[0,\frac{1+2r}{3+2r}]$,$\frac{\partial\Delta\pi_{R}^{CS}}{\partial\theta}\geq0$, $\Delta\pi_{R}^{CS}$ increases with $\theta$; if $\theta\in(\frac{1+2r}{3+2r},1]$, $\frac{\partial\Delta\pi_{R}^{CS}}{\partial\theta}<0$, $\Delta\pi_{R}^{CS}$ decreases with $\theta$. Let $\Delta\pi_{R}^{CS}=0$, we can get that $\theta=0$ or $\theta=\frac{1+2r}{2(1+r)}$. If $\theta\in[0,\frac{1+2r}{2(1+r)}]$, $\Delta\pi_{R}^{CS}\geq0$; if $\theta\in(\frac{1+2r}{2(1+r)},1]$, $\Delta\pi_{R}^{CS}<0$. According to the above, CS can bring additional profits to both the retailer and the supplier only when $0\leq\theta\leq\min\{\theta_{0},\frac{1+2r}{2(1+r)}\}$. In addition, we find that when $\theta=\frac{1+2r}{2(1+r)}$, $\Delta\pi_{S}^{CS}=-\frac{g^{2}\eta(1+r)(1+2r)(a-bc-bcr)^{2}}{8[2b\eta(1+r)-g^{2}]^{2}}<0$ which means $\theta_{0}<\frac{1+2r}{2(1+r)}$. The supplier and the retailer can reach an agreement on CS within $0\leq\theta\leq\theta_{0}$. □

**Proof of Proposition 3.**

The optimal operation decisions of players are determined by the backward induction approach. According to Equation (20), the Lagrange equation of the supplier's profit is:

Kuhn-Tucker conditions are as follows:

$\frac{\partial L}{\partial\Delta e^{PF}}=\frac{\partial L}{\partial w^{PF}}=0$; $\lambda(\Delta e^{PF}-\underline{e})=0$; $\lambda\geq0$.

If $\lambda=0$, the supplier's decisions do not affect by the carbon emission reduction standard. According to the first derivative, we have

$w^{PF}=\frac{(1+r-s)[\eta(a+bc+bcr-bcs-bm_{r}^{PF})-cg^{2}]}{2b\eta(1+r-s)-g^{2}}$ and $\Delta e^{PF}=\frac{g[a-bc(1+r-s)+bm_{r}^{PF}]}{2b\eta(1+r-s)-g^{2}}$. Submitting results to Equation (21), the retailer's profit function, we calculate the second derivative to know . Let $\frac{\partial\pi_{R}^{PF}}{\partial m_{r}^{PF}}=0$, $m_{r}^{PF*}=\frac{a-bc(1+r-s)}{2b}$ can be obtained. At this point, we can get $\Delta e^{PF*}$, $w^{PF*}$, $p^{PF*}$, and $s'<s\leq r$.

If $\lambda>0$, the supplier's decisions will be constrained by the carbon emission reduction standard, and $\Delta e^{PF*}=\underline{e}$. According to the first derivative, we have $w^{PF}=\frac{a+bc(1+r-s)+g\underline{e}-bm_{r}^{PF}}{2b}$. Submitting results to Equation (21), we calculate $\frac{\partial\pi_{R}^{PF}}{\partial m_{r}^{PF}}=0$ to know $m_{r}^{PF*}=\frac{a-bc-bcr+bcs+g\underline{e}}{2b}$. At this point, we can obtain $\Delta e^{PF*}$, $w^{PF*}$, $p^{PF*}$and $0\leq s\leq s'$. □

**Proof of Proposition 4.**

According to Proposition 3, if $s'<s\leq r$, i.e., $\Delta e^{PF*}=\frac{g[a-bc(1+r-s)]}{4b\eta(1+r-s)-2g^{2}}=e'\geq\underline{e}$, we can get $\frac{\partial\Delta e^{PF*}}{\partial s}=\frac{bg(2a\eta-cg^{2})}{2[2b\eta(1+r-s)-g^{2}]^{2}}>0$, the supplier's carbon emission reduction level increases with $s$. Besides, it is not difficult to find that $\Delta e^{PF*}=\Delta e^{*}$ when $s=0$. Therefore, $\Delta e^{PF*}\geq\Delta e^{*}$. If $0\leq s\leq s'$, the carbon emission reduction standard has a binding effect and $\Delta e^{PF*}=\underline{e}\geq e'$. It has also been confirmed that $e'\geq\Delta e^{*}$. Thus, there is always $\Delta e^{PF*}\geq\Delta e^{*}$ under PF. □

**Proof of Proposition 5.**

For the retailer: ① If $s'<s\leq r$, i.e., $\underline{e}<e'$, according to Equation (30), $\frac{\partial\pi_{R}^{PF*}}{\partial s}=\frac{[a-bc(1+r-s)]\eta\{ag^{2}+bc(1+r-s)[4b\eta(1+r-s)-3g^{2}]\}}{4[2b\eta(1+r-s)-g^{2}]^{2}}>0$. And $\pi_{R}^{PF*}=\pi_{R}^{*}$ when $s=0$. So we can get $\pi_{R}^{PF*}\geq\pi_{R}^{*}$. ② If $0\leq s\leq s'$, $\Delta e^{PF*}=\underline{e}>e'$, we analyze Equation (33) and know that $\pi_{R}^{PF*}$ increases with $\underline{e}$, $\pi_{R}^{PF*}-\pi_{R}^{*}>\frac{g^{2}[a-bc(1+r-s)]^{2}}{32b[2b\eta(1+r-s)-g^{2}]}>0$. So the retailer always gets a higher profit under PF regardless of how to set $\underline{e}$.

Similarly, for the supplier: ① If $s'<s\leq r$, i.e., $\underline{e}<e'$, according to Equation (29), $\frac{\partial\pi_{S}^{PF*}}{\partial s}=\frac{[a-bc(1+r-s)]\eta\{ag^{2}+bc(1+r-s)[4b\eta(1+r-s)-3g^{2}]\}}{8[2b\eta(1+r-s)-g^{2}]^{2}}>0$. And $\pi_{S}^{PF*}=\pi_{S}^{*}$ when $s=0$. So we can get $\pi_{S}^{PF*}\geq\pi_{S}^{*}$. ② If $0\leq s\leq1+r-\frac{ag+2g^{2}\underline{e}}{bcg+4b\eta\underline{e}}$, $\Delta e^{PF*}=\underline{e}>e'$, we analyze Equation (32) and know that $\frac{\partial^{2}\pi_{S}^{PF*}}{\partial\underline{e}^{2}}=\frac{2g^{2}-16b\eta(1+r-s)}{16b}<0$, that is, $\pi_{R}^{PF*}$ is concave in $\underline{e}$. Let $\frac{\partial\pi_{S}^{PF*}}{\partial\underline{e}}=0$, we can obtain $\underline{e}=\frac{g[a-bc(1+r-s)]}{8b\eta(1+r-s)-g^{2}}$. Because of $\frac{g[a-bc(1+r-s)]}{8b\eta(1+r-s)-g^{2}}<e'$, $\pi_{S}^{PF*}$ decrease with $\underline{e}$ when $\underline{e}>e'$. Suppose that $e^{PF}$ satisfies $\pi_{S}^{PF*}-\pi_{S}^{*}=0$ in the region $\underline{e}\in[\frac{g[a-bc(1+r-s)]}{8b\eta(1+r-s)-g^{2}},+\infty)$. If $e^{PF}\geq e'$, the condition that satisfies $\pi_{S}^{PF*}\geq\pi_{S}^{*}$ is $e'\leq e\leq e^{PF}$; if $e^{PF}<e'$, there is always $\pi_{S}^{PF*}<\pi_{S}^{*}$. Therefore, the supplier agrees to accept PF when $0\leq\underline{e}\leq\max\{e',e^{PF}\}$. With comprehensive consideration of the retailer and the supplier, the two sides can reach a consensus in $0\leq\underline{e}\leq\max\{e',e^{PF}\}$. □

**Proof of Proposition 6.**

If $s'<s\leq r$, $\frac{\partial\Delta e^{PF*}}{\partial s}>0$ (see Proposition 4). Let $\Delta e^{PF*}=\Delta e^{CS*}$, we can know

$s_{e}=\frac{[a-bc(1+r)][4b\eta(1+r)^{2}(1-\theta)-g^{2}]\theta}{4ab(1+r)\eta(1-\theta)-4b^{2}c(1+r)^{2}\eta(1-\theta)\theta-bcg^{2}[2+2r(1-\theta)-3\theta]}$.

When $s\geq\max\{s_{e},s'\}$, $\Delta e^{PF*}\geq\Delta e^{CS*}$ is satisfied. If $0\leq s\leq s'$, $\Delta e^{PF*}=\underline{e}>e'$. $\Delta e^{PF*}\geq\Delta e^{CS*}$ is still satisfied when $s\geq s_{e}$. To sum up, when $s\geq s_{e}$, there is always $\Delta e^{PF*}\geq\Delta e^{CS*}$. □

**Proof of Proposition 7.**

If $s\geq s'$, we have $\frac{\partial\pi_{S}^{PF*}}{\partial s}>0$ and $\frac{\partial\pi_{R}^{PF*}}{\partial s}>0$, when and $s_{S1}$, $s_{R1}$ is the unique value of $s$ that satisfies $\pi_{S}^{PF*}=\pi_{S}^{CS*}$, $\pi_{R}^{PF*}=\pi_{R}^{CS*}$ respectively. When $\max\{s_{S1},s_{R1},s'\}\leq s\leq r$, both the retailer and the supplier can get higher profits under PF. If $s<s'$, we also have $\frac{\partial\pi_{S}^{PF*}}{\partial s}>0$ and $\frac{\partial\pi_{R}^{PF*}}{\partial s}>0$, and $s_{S2}$, $s_{R2}$ is the unique value of $s$ that satisfies $\pi_{S}^{PF*}=\pi_{S}^{CS*}$, $\pi_{R}^{PF*}=\pi_{R}^{CS*}$ respectively. When $\max\{s_{S2},s_{R2}\}\leq s\leq s'$, two parties all prefer PF. Combining the above two cases, Proposition 7 can be obtained. □

**Proof of Proposition 8.**

This proposition is a continuation of Proposition 5. Proposition 5 has confirmed that when $s'<s\leq r$ (i.e., $0\leq\underline{e}<e'$), the carbon reduction standard does not act as a constraint, and that $\pi_{R}^{PF*}\geq\pi_{R}^{*}$ and $\pi_{S}^{PF*}\geq\pi_{S}^{*}$ are constant. Therefore, for any setting that satisfies $0\leq\underline{e}*<e'$ is optional for the retailer. When $0\leq s\leq s'$ (i.e., $e'\leq\underline{e}\leq r$), there is $\pi_{R}^{PF*}\geq\pi_{R}^{*}$ and $\frac{\partial\pi_{R}^{PF*}}{\partial\underline{e}}>0$, so the retailer will set the carbon reduction standard as high as possible. However, for the supplier, there is $\pi_{S}^{PF*}\geq\pi_{S}^{*}$ only at $\underline{e}\leq e^{PF}$, where $e^{PF}$ is a valid solution for $\pi_{S}^{PF*}-\pi_{S}^{*}=0$. Therefore, it is now only necessary to determine the relationship between the magnitudes of $e^{PF}$ and $e'$. If $e'\leq e^{PF}$, then $\underline{e}*=e^{PF}$; if $e'>e^{PF}$, then $0\leq\underline{e}*<e'$. Since $\frac{\partial\pi_{S}^{PF*}}{\partial\underline{e}}<0$ , when $\underline{e}=e'$ , if $\pi_{S}^{PF*}-\pi_{S}^{*}|_{\underline{e}=e'}\geq0$ , we have $e'\leq e^{PF}$ ; if $\pi_{S}^{PF*}-\pi_{S}^{*}|_{\underline{e}=e'}<0$ , we have $e'>e^{PF}$ . Let $\hat{s}$ be a valid solution that satisfies $\pi_{S}^{PF*}-\pi_{S}^{*}|_{\underline{e}=e'}=0$. According to $\frac{\partial\pi_{S}^{PF*}}{\partial s}>0$, it is obtained that when $0\leq s<\hat{s}$, we have$\pi_{S}^{PF*}<\pi_{S}^{*}$ , $\underline{e}*$ only needs to satisfy $0\leq\underline{e}*<e'$; when $\hat{s}\leq s<s'$, we have$\pi_{S}^{PF*}\geq\pi_{S}^{*}$ , $\underline{e}*=e^{PF}$ . In addition, considering the constraint of $0\leq s<r$, let $\bar{s}$ be a valid solution satisfying $s'-r|_{\underline{e}=e^{PF}}=0$. Combining with the case of $s'<s\leq r$, we can get that when $0\leq s\leq\hat{s}$ or $\bar{s}\leq s\leq r$, $\underline{e}*$ only needs to satisfy $0\leq\underline{e}*<e'$; when $\hat{s}<s<\bar{s}$, $\underline{e}*=e^{PF}$. □
