# Supplementary material for: Internal incentives for carbon emission reduction in a capital-constrained supply chain: A financial perspective
Source: PLoS One. 2023 Jul 6;18(7):e0287823. doi: 10.1371/journal.pone.0287823 (PMC10325093; doi:10.1371/journal.pone.0287823)
Supplement: S1 File — (DOCX) [file pone.0287823.s002.docx]

**Data description of numerical examples**

The conclusions presented in the article are primarily derived from analytical formulations, but numerical examples are given to visually illustrate the relevant findings. Based on the model analysis, we make assumption assignments to relevant parameters. The setting of the data is derived from considerations of reality and mainly includes several aspects:

(1) The sales profit of both the retailer and the supplier must be non-negative.

(2) The market demand must be non-negative.

(3) Under CS, the retailer's share of carbon abatement costs must not be negative nor greater than 1.

(4) Under PF, the preferential margin of interest rate cannot be negative and cannot exceed the initial interest rate.

Numerous data sets meet the aforementioned real constraints, and there can be multiple choices for parameter settings. However, due to space limitations, this analysis in this paper is based on the following set of data:

The initial market size: $a=50$;

The production cost per unit product: $c=9$;

The sensitivity of market demand to the retail price: $b=3$;

The sensitivity of market demand to carbon emission reduction level: $g=3$;

The carbon emission reduction cost parameter: $\eta=6$;

The base financing rate: $r=0.3$;

The cost sharing ratio satisfies $\theta\in[0,1]$;

The preferential margin of the interest rate satisfies $s\in[0,0.3]$.
